# Patient Companionship in a Movement Disorders Clinic: Outside Assistance Inside the Office

**DOI:** 10.3389/fneur.2019.00182

**Published:** 2019-03-05

**Authors:** Ana Vives-Rodriguez, Daniel Trujillo Diaz, Elan D. Louis

**Affiliations:** ^1^Division of Movement Disorders, Department of Neurology, Yale School of Medicine, Yale University, New Haven, CT, United States; ^2^Center for Neuroepidemiology and Clinical Neurological Research, Yale School of Medicine, Yale University, New Haven, CT, United States; ^3^Department of Chronic Disease Epidemiology, Yale School of Public Health, Yale University, New Haven, CT, United States

**Keywords:** movement disorders, visit companions, caregiver, Parkinson's disease, essential tremor

## Abstract

**Objective:** We (1) report whether a companion (i.e., spouse, relative, aide) accompanied our consecutive outpatients with a range of movement disorders, (2) identified the set of patient characteristics that was associated with the need for a visit companion, and (3) characterized the role(s) of these companions during the visit. Our overarching goals were to further understand patient needs and the extent of their support networks, and to enrich the clinician-patient interface.

**Methods:** Two-hundred consecutive patients were enrolled from the Movement Disorders Clinic at Yale School of Medicine. We noted whether patients were accompanied by another person during the visit and documented the role of the visit companion during the encounter.

**Results:** One-hundred-twenty-eight of 200 patients (64.0%) brought a companion, with these being spouses (44.8%), adult children (24.1%) or an aide, nurse or social worker (14.5%). Patients who were unemployed (odds ratio [OR] = 5.32, *p* = 0.019), had a diagnosis of Parkinson's disease or other Parkinsonian syndromes (OR = 10.61, *p* = 0.001), or were dependent in any instrumental activities of daily living (iADLs) (OR = 4.99, *p* = 0.005) or basic activities of daily living (bADLs) (OR = 5.81, *p* = 0.02), had increased odds of presenting to the clinical visit with a visit companion. Visit companions' main roles involved communication (86.7%) and transportation (84.4%).

**Conclusion:** Visit companions were commonly present during movement disorders outpatient visits–two-thirds of patients were accompanied. A number of factors increased the odds of requiring such a companion by 4- or 5-fold.

## Introduction

Movement disorders (e.g., Parkinson's disease [PD], essential tremor [ET]) are common neurological conditions that often affect motor independence (1, 2) and may also be accompanied by cognitive difficulties (3) and behavioral comorbidities (4, 5). Therefore, patients with movement disorders are considered a vulnerable population whose quality of life is often reduced by their burden of disease and disease-associated comorbidities (6–8).

Several studies have focused on the importance of family participation in patient care (5–8). For example, family members are partners in preventing hospital errors and in improving surveillance of adverse effects (9). However, studies of family involvement during outpatient medical visits are few in number and mainly oriented toward patients in geriatric and primary care settings, (10, 11) although some work has been done in neurology settings (12–15). These studies suggest an important role of family members both in enhancing patient-physician communication and in serving as agents of patient satisfaction with physician care (16).

There is a curious gap in knowledge. There are a limited number of studies of family participation in patients with neurological disorders (12–15). How often are such patients accompanied to their outpatient visit and what role(s) do companions play during these visits? In movement disorders in particular, motor, cognitive, and behavioral issues are quite common (1–5, 8). However, these issues are patchily distributed across these disorders (e.g., more common in some disorders than others, and dependent on the duration and stage of each disorder), leading to a complex mélange of patient conditions and needs. *A priori*, we hypothesized that older patients, patients with cognitive impairment, patients with functional dependency, and patients with certain disorders (e.g., PD) would most frequently be accompanied during their encounter. However, we had no *a priori* sense of the percentage of patients with each condition who might require a companion.

In this study of 200 outpatient movement disorder visits, we aimed to (1) report whether a companion (i.e., spouse, relative, aide) accompanied our patients with a range of movement disorders, (2) identify the set of patient characteristics that was associated with the need for a visit companion, and (3) characterize the role(s) of these companions during the visit. Our overarching goals were to contribute to information regarding patient needs, their health care behaviors, and their support networks, and to enrich the clinician-patient interface.

## Materials and Methods

### Study Design

Patients were consecutively recruited by AV-R, a movement disorders fellow, from September–December 2017 from the Movement Disorders Clinic at Yale School of Medicine. AV-R had an average of four half-day clinics per week with four different movement disorder neurologists (including EDL). The study was reviewed and approved by the Yale University Institutional Review Board (IRB) (IRB #2000024723). The Yale IRB committee approved a written informed consent waiver. Subjects provided informed assent and were permitted to withdraw from participation at any time.

At the end of the clinic visit, demographic and clinical information were collected directly from the patient and their clinical records, including age, gender, distance from our medical center, type of insurance (e.g., Medicare, Medicaid), median annual income in county of residence, ethnicity, visit type (e.g., routine visit, botulinum toxin clinic), initial, or follow up visit, number of canceled medical visits in previous year, diagnosis, patient mobility, and functionality/dependence, employment status, presence of a prior diagnosis of mild cognitive impairment or dementia (by history and chart review), whether or not the patient was accompanied to their appointment by another person, and if so, that person's relationship to the patient. A visit companion was defined as a person 18 years or older who came to the clinic visit with the patient.

Patient mobility was categorized based on how the patient entered the examination room (walking independently, using a cane, using a walker, or using a wheelchair). Patients were categorized as dependent in instrumental activities of daily living (iADLs) if they required assistance with one or more of the following activities: grocery shopping, household finances, driving/using public transportation, household chores, using the telephone, or taking medication. Patients were categorized as dependent in basic activities of daily living (bADLs) if they required assistance with one or more of the following activities: eating, bathing, getting dressed, toileting, and personal mobility (17, 18).

AV-R documented the roles of the visit companion during the encounter and categorized them into one or more of the following: (1) transportation, if the companion drove or picked the patient up in order to bring him/her to the appointment; (2) communication, if the companion provided medication history, gave additional medical history or if the companion directed the conversation for the patient; (3) moral support, if the companion provided reassurance after a disclosure of the diagnosis or re-framed difficult questions or statements for the patient; and (4) physical assistance, if the companion helped the patient get in/out of the chair or if the companion wheeled the patient into the office.

### Sample Size Determination and Data Analysis

Patients were enrolled consecutively until the targeted sample of 200 patients was reached. There were no refusals. Sample size was calculated according to the method proposed by Peduzzi et al. (19, 20) which is based on the expected proportion for the primary outcome and the number of variables that need to be accounted for in regression models during the analysis of data. Based on the most common movement disorders treated in our clinic and the age of our patients, we expected that the proportion of accompanied patients to be similar to that reported in geriatric outpatient encounters (~40%) (11, 16, 21). We expected to include eight independent variables in the final multivariate logistic regression model (described below).

Analyses were performed in Stata (Version IC 15.1). To facilitate data analysis, diagnoses were collapsed into 5 categories: (1) PD and other parkinsonian syndromes, (2) dystonia, (3) ET, (4) other degenerative disorders or disorders with spasticity/gait impairment (e.g., cerebellar ataxias and Huntington's disease), and (5) other non-degenerative disorders without gait impairment (e.g., tardive dyskinesia and restless legs syndrome).

We first reported the proportion of patients who had a visit companion. Then, bivariate analyses were performed using Mann-Whitney and chi-square tests to evaluate differences between patients for whom a visit companion was present vs. patients for whom a visit companion was not present. A series of bivariate logistic regression analyses assessed the association between clinical variables and the presence vs. absence of a visit companion (dependent variable), resulting in odds ratios (ORs), 95% confidence intervals (CIs), and *p*-values. Significance was set at *p* < 0.05. Finally, after identifying statistically significant variables from the bivariate analysis, multivariate logistic regression models were constructed to determine which factors were independently associated with visit companionship.

## Results

Our targeted enrollment of 200 patients was met after 3 months (Table 1). The median age was 68.0 years (inter-quartile range [IQR] = 58.0–74.5) and both genders were nearly equally represented. The three most frequent diagnoses were PD and other parkinsonian syndromes (*n* = 101, 50.5%), dystonia (*n* = 25, 12.5%), and ET (*n* = 16, 8.0%), accounting for more than two-thirds of enrollees. Median age for these diagnoses were 71 years (IQR = 66–76) for PD, 58 years (IQR = 51–64) for dystonia and 70.5 years (IQR = 66–74) for ET (Kruskal Wallis; *p* = 0.001). Only the dystonia group had a significant difference in age compared to ET and PD cases (Mann Whitney with Bonferroni correction: ET vs. dystonia *p* = 0.004; ET vs. PD *p* = 0.43; dystonia vs. PD *p* = < 0.001). Eight percent of patients reported a previous diagnosis of mild cognitive impairment or dementia. Two-thirds (64.0%) came to the visit walking independently without the use of a cane or walker and 53.0% were independent for both iADL and bADL.

**Table 1:** Clinical characteristics of 200 patients.

**Characteristics**	**Data**
Age in years	Median: 68.0 (IQR: 58.0–74.5)
Female gender	96 (48.0)
Distance from home to our medical center (km)	Median: 27.5 (IQR: 12.1–57.5)
TYPE OF INSURANCE^*^	
Medicare	77 (39.3)
Medicaid	14 (7.1)
Blue cross blue shield	7 (3.6)
Self pay	3 (1.5)
Other	41 (20.9)
Multiple	54 (27.6)
Median annual income in county of residence	$64,872
**ETHNICITY**	
Caucasian	160 (80.0)
Hispanic	21 (10.5)
Black	15 (7.5)
Asian	4 (2.0)
**WORK STATUS**	
Retired	131 (65.5)
Employed	39 (19.5)
Unemployed	28 (14.0)
Student	2 (1.0)
**VISIT TYPE**	
Routine visit to clinic	159 (79.5)
Botulinum toxin clinic	27 (13.5)
Deep brain stimulation clinic	14 (7.0)
**INITIAL OR FOLLOW UP VISIT**	
Initial visit	51 (25.5)
Follow up visit	149 (74.5)
Median number of canceled medical visits in previous year	4
**DIAGNOSIS**	
Parkinson's disease and other parkinsonian syndromes	101 (50.5)
Dystonia	25 (12.5)
Essential tremor	16 (8.0)
Other degenerative disorders or disorders with spasticity or gait impairment	24 (12.0)
Other non-degenerative disorders without gait impairment	34 (17.0)
**PRIOR DIAGNOSIS OF COGNITIVE IMPAIRMENT**	
None	184 (92.0)
Mild cognitive impairment	7 (3.5)
Dementia	8 (4.0)
Intellectual disability	1 (0.5)
**PATIENT MOBILITY**	
Independent	128 (64.0)
Cane	24 (12.0)
Walker	10 (5.0)
Wheelchair	38 (19.0)
**FUNCTIONALITY**	
Independent	106 (53.0)
Dependent in iADLs	43 (21.5)
Dependent in bADLs	51 (25.5)
**VISIT COMPANION**	
Present	128 (64.0)
Absent	72 (36.0)
**NUMBER OF COMPANIONS**	
1	113 (56.5)
2	13 (6.5)
3	2 (1.0)
**RELATIONSHIP OF VISIT COMPANION TO PATIENT (*****n*** **= 145)**	
Spouse	65 (44.8)
Adult Child	35 (24.1)
Other relative/friend (e.g., aunt, nephew)	11 (7.6)
Parent	7 (4.8)
Sibling	6 (4.1)
Aide, nurse or social worker	21 (14.5)
**VISIT COMPANION ROLE (*****n*** **= 128)**	
Communication	111 (86.7)
Transportation	108 (84.4)
Physical assistance	39 (30.5)
Moral support	18 (14.1)

*Values represent number (percentage) or medians with interquartile ranges; IQR, interquartile range; iADLs, instrumental activities of daily living; bADLs, basic activities of daily living*.

* *Data missing on several subjects*.

One-hundred-twenty-eight of 200 patients (64.0%) brought a visit companion. The total number of visit companions was 145 (i.e., some patients had more than one visit companion). Spouses were the most frequent type of companion (65 of 145, 44.8%), followed by adult children (24.1%), or aides, nurses, or social workers (14.5%). In two cases, the visit companion provided transportation but stayed in the waiting area during the encounter. Among companions who were family members (*n* = 124), 86 (69.4%) were female and 38 (30.6%) were male.

Among 128 patients who came in with a visit companion, the companions' main roles involved communication (111 of 128, 86.7%) and transportation (84.4%). Only 30.5% of patients who had a companion required physical assistance from the companion during the encounter.

Accompanied vs. unaccompanied patients differed with respect to numerous demographic and clinical characteristics. Accompanied patients were on average older, and were more often retired, parkinsonian, impaired in cognition, impaired in mobility, and functionally dependent (Table 2). They did not differ in numerous respects (e.g., gender, distance from medical center, type of insurance, median annual income in county of residence, median number of canceled medical visits in the previous year).

**Table 2:** Comparison of clinical characteristics of accompanied vs. unaccompanied patients.

**Clinical characteristics of patient**	**Patients for whom a visit companion was present *n* = 128**	**Patients for whom a visit companion was not present *n* = 72**	***p*-value**
Age in years	70.5 (IQR:64.0–76.0)	63.0 (IQR:54.0–71.0)	<0.001^a^
Female gender	62 (48.4)	34 (47.2)	0.88^b^
Distance from home to our medical center (km)	28.2 (IQR: 14.5–58.6)	25.3 (IQR: 4.2–54.2)	0.10^a^
TYPE OF INSURANCE*			
Medicare	56 (45.2)	21 (29.2)	0.22^b^
Medicaid	8 (4.1)	6 (8.3)	
Blue cross blue shield	4 (3.2)	3 (4.2)	
Self pay	2 (1.6)	1 (1.4)	
Other	20 (16.1)	21 (29.2)	
Multiple	34 (27.4)	20 (27.8)	
Median annual income in county of residence	$64,872	$64,872	1.00^a^
**ETHNICITY**			
Caucasian	101 (63.1)	59 (36.9)	0.48^b^
Hispanic	14 (66.7)	7 (33.3)	
Black	9 (60.0)	6 (40.0)	
Asian	4 (100)	0 (0.0)	
**WORK STATUS**			
Retired	95 (72.5)	36 (27.5)	<0.001^b^
Unemployed	21 (75.0)	7 (25.0)	
Employed	11 (28.2)	28 (71.8)	
Student	1 (50.0)	1 (50.0)	
**VISIT TYPE**			
Routine visit to clinic	102 (64.2)	57 (35.8)	0.10^b^
Botulinum toxin clinic	14 (51.9)	13 (48.1)	
Deep brain stimulation clinic	12 (85.7)	2 (14.3)	
**INITIAL OR FOLLOW UP VISIT**			
Initial visit	33 (64.7)	18 (35.3)	0.90^b^
Follow up visit	95 (63.8)	54 (36.2)	
Median number of canceled medical visits in previous year	4	3.5	0.90^a^
**DIAGNOSIS**			
Parkinson's disease and other parkinsonian syndromes	82 (81.2)	19 (18.8)	<0.001^b^
Dystonia	8 (32.0)	17 (68.0)	
Essential tremor	7 (43.8)	9 (56.2)	
Other degenerative disorders or disorders with spasticity or gait impairment	13 (54.2)	11 (45.8)	
Other non-degenerative without gait impairment	18 (52.9)	16 (47.1)	
**PRIOR DIAGNOSIS OF COGNITIVE IMPAIRMENT**			
None	113 (61.4)	71 (38.6)	0.04^b^
Mild cognitive impairment	6 (85.7)	1 (14.3)	
Dementia	8 (100)		
Intellectual disability	1 (100)		
**PATIENT MOBILITY**			
Independent	69 (53.9)	59 (46.1)	<0.001^b^
Cane	14 (58.3)	10 (41.7)	
Walker	10 (76.9)	3 (23.1)	
Wheelchair	35 (100)	0 (0.0)	
**FUNCTIONALITY**			
Independent	45 (42.5)	61 (57.5)	<0.001^b^
Dependent in iADLs	36 (83.7)	7 (16.3)	
Dependent in bADLs	47 (92.2)	4 (7.8)	

*Values represent number (percentage) or medians with interquartile ranges. All percentages are row percentages rather than column percentages. IQR, interquartile range; iADLs, instrumental activities of daily living; bADLs, basic activities of daily living*.

a *Mann Whitney test*.

b *Chi square test*.

Of the 16 patients with prior diagnosis of cognitive impairment, 15 (93.8%) were accompanied. Of the 101 patients with a diagnosis of parkinsonism, 81.2% were accompanied in contrast to patients with dystonia and ET who were less frequently accompanied (32.0% [*p* < 0.001] and 43.8% [*p* = 0.001], respectively). All patients in a wheelchair had a companion during the visit as compared to 76.9% of patients with a walker, 58.3% of patients with a cane, and 53.9% of patients who were independent with respect to mobility (*p* < 0.001). Of the 51 patients who required assistance with bADL, 92.2% were accompanied. In contrast, only 42.5% of patients who were fully independent in ADLs were accompanied to the visit (*p* < 0.001).

In a series of bivariate logistic regression analyses (Table 3), older age, a diagnosis of parkinsonism, and a presence of any cognitive impairment were associated with higher odds of having a companion during the visit. Retired and unemployed patients were also more likely to be accompanied to the visit (reference group = employed patients). Patients who were dependent with iADLs, bADLs (reference group = independence in daily activities), and patients who required a walker or a wheelchair (reference group = independence in ambulation or use of a cane) had increased odds of having a visit companion present. No association was documented for a diagnosis of ET, other degenerative and other non-degenerative disorders, traveling distance, patient's ethnicity, or visit type.

**Table 3:** Association between a presence of a visit companion and patients' clinical variables.

**Clinical variables**	**Odds ratio**	**95% CI**	***p*-value**
Age in years	1.04	1.02–1.07	0.001
**GENDER**			
Female	1.05	0.59–1.87	0.87
Male (reference group)	1.00		
Distance from home to our medical center (km)	1.01	0.99–1.02	0.12
**ETHNICITY**			
Hispanic	1.16	0.45–3.06	0.75
Black	0.88	0.30–2.58	0.81
Caucasian (reference group)	1.00		
**WORK STATUS**			
Unemployed	7.64	2.53–23.03	<0.001
Retired	6.72	3.03–14.89	<0.001
Student	2.55	0.15–44.37	0.52
Employed (reference group)	1.00		
**INITIAL OR FOLLOW UP VISIT**			
Initial	0.96	0.49–1.86	0.90
Follow up (reference group)	1.00		
**DIAGNOSIS**			
Parkinsonism	9.17	3.45–24.37	<0.001
Other degenerative disorders	2.51	0.79–8.03	0.12
Other non-degenerative disorders	2.39	0.81–7.02	0.11
Essential tremor	1.65	0.45–6.05	0.45
Dystonia (reference group)	1.00		
**PRIOR DIAGNOSIS OF COGNITIVE IMPAIRMENT**			
MCI or dementia	9.42	1.22–72.91	0.03
None (reference group)	1.00		
**PATIENT MOBILITY**			
Use of a walker or wheelchair	12.47	3.71–41.88	<0.001
Independent or use of cane (reference group)	1.00		
**FUNCTIONALITY**			
Dependent in iADLs	6.97	2.84–17.09	<0.001
Dependent in bADLs	15.93	5.35–47.42	<0.001
Independent (reference group)	1.00		

*CI, confidence interval; iADLs, instrumental activities of daily living; bADLs, basic activities of daily living. Each row presents data of a bivariate logistic regression analysis in which visit companion (present vs. absent) was the dependent variable*.

We performed a multivariate logistic regression analysis including age, gender, work status, diagnosis, presence of cognitive impairment, patient mobility and functionality, and found that only patients who were unemployed (OR = 5.32, *p* = 0.019, *CI*: 1.31–21.61), had a diagnosis of PD or other parkinsonian syndromes (OR = 10.61, *p* = 0.001, *CI*: 2.68–41.97), or were dependent in any iADLs (OR = 4.99, *p* = 0.005, *CI*: 1.63–15.25) or dependent in any bADLs (OR = 5.81, *p* = 0.02, *CI*: 1.29–26.04), had increased odds of presenting to the clinical visit with a companion. No association was documented for the use of a walker or a wheelchair for mobility, but a trend was seen (OR = 4.14, *p* = 0.08, *CI*: 0.83–20.69).

## Discussion

Previous studies performed in primary care and geriatric clinics have reported that 30–50% of patients are accompanied during outpatient visits (10, 11, 21). Though our sample was younger than those of previously described geriatric cohorts [median age of this cohort: 68 years vs. 74–78.5 years in previous studies (11, 16, 22), we documented a higher proportion of patients who brought companions (64.0%). This higher proportion may be due to the progressive nature and comorbidities associated with movement disorders. The prevalence varied considerably across the different movement disorders, from 32.0% in dystonia patients to 81.2% in PD patients. Even in ET patients, which is a disorder that progresses slowly and does not compromise mobility to a marked degree in most patients, the prevalence was more than 40%.

In our study, patients were primarily accompanied by family members (spouse and adult children). As in prior studies, we documented a female predominance in patients' companions (69.4%). This could be related to the fact that women are still described as the predominant care providers for family members with chronic medical conditions or disabilities (23). However, in the parkinsonian group, which had the highest prevalence of visit companions, there was a male predominance among patients who were accompanied (58 males vs. 32 females). This could have potentially shifted the companions' gender in the sample to a female predominance (i.e., their spouses).

As anticipated, the presence of a visit companion was associated with a diagnosis of PD or parkinsonian syndromes and functional impairment. Specifically, in multivariate analyses, a diagnosis of PD or other parkinsonian syndromes was associated with more than ten times increased odds of being accompanied to the visit. Parkinsonian syndromes are progressive neurodegenerative disorders that not only affect mobility but also manifest with an array of non-motor symptoms, including depression, apathy, dysautonomia, and cognitive decline. These symptoms can significantly affect quality of life (7, 24).

Furthermore, functional impairment was independently associated with the presence of a visit companion, with a higher association in patients who were dependent in bADLs than in iADLs. Surprisingly, impaired patient mobility did not achieve statistical significance in predicting patient companionship. Though this could simply be due to study power and sample size, one might also infer that patient disability (based on ADLs) plays a more important role among patients requiring companionship.

Unemployed patients had a higher prevalence of companions as well. Upon review of the cases, these corresponded to patients with significant psychiatric or neurological disorders that complicated their ability to work, and many required government assistance. This suggests that the presence of a visit companion during clinical visits could be a marker associated with social risk and functional dependence.

We had hypothesized that older patients as well as patients with cognitive impairment would most frequently be accompanied during their encounters. Indeed, in initial analyses (Tables 2, 3), both variables were significantly associated with presence of a visit companion. However, in multivariate models, neither variable remained. For cognitive impairment, this could be related to the small proportion of patients with a prior diagnosis of cognitive impairment in our cohort (*n* = 16 or 8.0%). In some ways, it is not surprising that age did not remain significant in the final model either, as it is probably not age itself that is responsible for the need for a visit companion, but rather, *age-associated* issues such as parkinsonism and difficulty with ADLs, which did remain in the model.

During the clinical encounters, visit companions were most frequently involved with transportation logistics and communication. These aspects appeared to be more important during these visits than physical assistance. As previously described in geriatric populations, visit companions are potential facilitators during clinical visits, actively engaging in the exchange of health information (16). In movement disorders, their presence becomes vital, especially among more vulnerable, and functionally impaired patients.

The data presented here contribute to our understanding of patient needs, their health care behaviors, and their support networks, with the over-arching goal to enrich the clinician-patient interface. The fact that some patients utilize visit companions suggests that they are a vulnerable group, requiring the additional assistance of such companions. The presence of a companion should alert the physician to this fact and forewarn them to spend additional time with that patient to explain treatment plans and to ensure that such plans are practical given limitations in the patient's capacities and circumstances.

To our knowledge, our study is the first to analyze the presence of visit companions during outpatient care in patients with a range of movement disorders. With our large sample size, we managed to enroll the most common movement disorders encountered in clinic and we collected data and were able to analyze a broad range of clinical, demographic, and social variables in the analysis.

A main limitation of this study is that only one clinician oversaw the assignment of the companion's role. Even though we defined specific criteria *a priori* to avoid subjectivity, our study design could have influenced our results. Second, patients did not provide insight into companion roles, or lack thereof. Therefore, the complexity of the physician-patient-companion relationship was not fully explored; other studies have taken additional steps to define and expand on the role of the companion more fully (12). Third, some variables were determined through chart review (e.g., cognitive impairment) and may not accurately reflect patients' status and other variables were not directly assessed (e.g., medical literacy). Fourth, a range of other social factors could have influenced patient behaviors and use of a visit companion; (14) future studies should explore these issues. Among variables that we did not consider were education and the availability of family members. Fifth, although the sample size was 200, in some cells, the numbers were small, and a larger sample size would have alleviated this issue; the use of Bayesian approaches to the data would also be of value in future studies. Finally, we did not explore how visit companions improved the quality of care. In other words, this was a foundational study of the frequency with which patients used a visit companion and the clinical correlates of that use. This was not a study of the utility of using such a companion or the effects that such companions had on care (i.e., this was not an outcomes study). Future longitudinal studies could review the prognostic value of companions in movement disorders clinics by documenting the association between absence of such companions and medication intake errors, lower treatment adherence, and loss to follow-up.

## Conclusion

In this cohort of movement disorders patients, the prevalence of visit companions was higher than previously described in other geriatric populations. Two-thirds of patients required such a companion. The presence of a visit companion was independently associated with a diagnosis of PD or other parkinsonian syndromes, unemployment, and functional impairment. Companions' role during the encounters focused on enhancing communication and assisting with patients' transportation. Several of these factors increased the odds of requiring such a companion by 4- or 5-fold. These data highlight the potential value of visit companions during neurological visits, especially among more vulnerable and functionally impaired patients.

## Disclosure

EL receives/has received research support from NIH/NINDS.

## Author Contributions

AV-R: drafting/revising the manuscript for content, including medical writing for content, study design, analysis and interpretation of data. DT: drafting/revising the manuscript for content, including medical writing for content, analysis or interpretation of data. EL: drafting/revising the manuscript for content, including medical writing for content, study concept and design, analysis and interpretation of data.

### Conflict of Interest Statement

The authors declare that the research was conducted in the absence of any commercial or financial relationships that could be construed as a potential conflict of interest.
